# Junior-to-senior transition in elite female football: identifying predominant sources of stress among junior players from both player and coach perspectives

**DOI:** 10.3389/fpsyg.2025.1621559

**Published:** 2025-08-21

**Authors:** Stian Aa Selbekk, Marthe Sofie Lilleengen, Janita Stålesen, Daniel Ransom, Stig Arve Sæther

**Affiliations:** ^1^Department of Sociology and Political Science, Norwegian University of Science and Technology (NTNU), Trondheim, Norway; ^2^Manchester United Football Club, Manchester, United Kingdom

**Keywords:** stressors, talent development, professional football, junior to senior, life balance

## Abstract

**Introduction:**

The purpose of this case study is to gain insight into and a deeper understanding of the predominant sources of stress during the junior-to-senior transition experienced by current and former female junior players and their male coaches from both the junior and senior teams.

**Methods:**

All three groups of participants were affiliated with the same Norwegian professional football club. In total, we conducted semistructured interviews with 10 female players: five junior elite players (mean age 16.8 years, SD = 0.96) and five senior elite players (mean age 23.4 years, SD = 2.01). In addition, four male coaches from the junior and senior teams (mean age 32.5 years, SD = 3.84) were interviewed. We conducted a thematic analysis of the interviews.

**Results and discussion:**

The findings revealed that the players experienced several sport stressors. Among the sport stressors, disparities in performance levels between the junior and senior teams, especially during matches, and the higher expectations and demands from their coaches were prominent. Among the non-sport stressors, the struggle to balance football with social life outside the sport was prominent. Since education was the elite female players’ backup plan, the club and school collaboration was considered important. Taken together, the results indicate a need for facilitating athletes’ daily lives to support and smooth their transition. On a broader level, this study contributes insights into the junior-to-senior transition in women’s football, an area that remains underrepresented in the research literature.

## 1 Introduction

The literature outlines the transition from junior to senior elite football as a complex process with several facets that can challenge and excite athletes (Drew et al., 2019). However, research on the junior-to-senior transition in women’s football is scarce. The few existing studies on female players have found that managing the balance between football, school, and social life is the most prominent challenge in the junior-to-senior transition (Andersson and Barker-Ruchti, 2018; Gledhill and Harwood, 2015). While these findings are in line with research on male players, it is proposed that female players experience these challenges to a greater extent due to the economic situation in female football (Bjerksæter and Lagestad, 2022). In a nation such as Norway, Women’s football has traditionally had a low status compared to men’s football among sponsors, media, supporters, and people in general, despite over one hundred thousand active female football players [Norges Fotballforbund (Norwegian Football Federation), 2024], making it the sport with the highest participation rate among girls and women. There has also been an increase in interest, attention, and investment at the elite level (Toppserien, 2025), and an increased professionalization with the introduction of professional days (working days with full focus on football). In addition to a reduction in the number of teams in the semi-professional league, to heighten the performance level of the league (Sæther et al., 2025). However, despite this increased professionalization—where most of the players at the elite level have professional contracts—most do not have full-time employment as players. Thus, several junior and senior players need to combine their football careers with other jobs or studies, which makes these players’ everyday life more demanding and stressful. Consequently, there are obvious differences between female and male players in their experience of the junior-to-senior transition in Norwegian football.

Although studies on male players might give some insights into the stressors experienced during the junior-to-senior transition (Andersson and Barker-Ruchti, 2018; Gledhill and Harwood, 2015), the unique experiences of female players need to be more represented in the literature to extend culturally specific understanding of these players (Stambulova and Ryba, 2014). One of the few studies on female players and the junior-to-senior transition was conducted by McGreary et al. (2021), who examined both junior and senior players within the same club. They found that the junior players appeared to have a different experience compared to the senior players, with the difference in experience attributed to the increased professionalization of female football (McGreary et al., 2021). Thus, junior players today might experience stressors to a greater extent than earlier. In addition, at the core of the handling of the junior-to-senior transition are the psychological aspects of the transition (Fortin-Guichard et al., 2022) and, especially, stressors such as sport and non-sport stressors (Drew et al., 2019). Therefore, many studies have adapted a holistic approach to grasp both sport and non-sport stressors that occur during this transition. Research on male football players has found that several factors act as stressors during the junior-to-senior transition, including pressure to perform at a higher level (Rye et al., 2022), integrating into a new environment (Kerdijk et al., 2016), balancing football and social life (Solhaug et al., 2021), competition with teammates (Platvoet et al., 2020), combining football with school (Sæther et al., 2022), selection and contract situations (Swainston et al., 2020), and impressing senior players during training and matches (Hem et al., 2022). In summary, these challenges highlight an obvious need for a holistic approach. The transition normally comes with a set of specific demands that athletes have to master to successfully continue their athletic development (Stambulova et al., 2009). Whether an athlete manages to master the transition depends on the athlete’s ability to balance resources and barriers. At the core of these challenges is how players handle the stressors associated with the junior-to-senior transition (Rye et al., 2022; Sæther et al., 2025).

Inspired by the study conducted by McGreary et al. (2021), our study draws on perspectives from both current junior players undergoing the transition and former junior players who have successfully completed the transition. In addition, as a further step, we sought to include the coaches’ perspective to incorporate their experience of how current and former players handle the stressors experienced during the junior-to-senior transition. Our aim was therefore to contribute insights into junior players’ experiences of sport and non-sport stressors during the junior-to-senior transition, based on interviews with both current and former junior athletes, as well as their coaches from both junior and senior teams.

## 2 Methods

### 2.1 Research design

The current study departed, based on the main aim of the study, from a qualitative approach grounded in a social interactionist ontology and adopted an interpretivist approach (Markula and Silk, 2011). We focused on the everyday interactions between individuals, examining how these interactions are interpreted and reinterpreted as individuals make sense of and adapt to their social environments. Furthermore, we highlight that multiple realities exist and that social reality is a subjective experience, thereby adopting a constructionist approach (Baxter and Jack, 2008). This study also highlights the need for letting the participants construct their reality within their contexts. In the present study, we aimed to understand how female junior players experience the junior-to-senior transition and which stressors they consider challenging in this transition. The data were collected using semi-structured interviews and analyzed using reflexive thematic analysis (Braun and Clarke, 2022). This study is part of a larger project on female athletes and their road to elite sports. In addition to focusing on players’ experiences, we also wanted to include their coaches and how and which stressors they consider to impact female players during the junior-to-senior transition. The rationale for including these three groups of participants was based on the potential differences between current and former players who had gone through the junior-to-senior transition, as well as the lack of previous studies. In addition, few studies have explored coaches’ experiences of players’ junior-to-senior transitions.

### 2.2 Participants

To gain an in-depth understanding of junior elite players’ (current and former) and male coaches’ perceptions of stressors related to the transition from junior to senior level, participants were recruited from a single football club, playing at the highest junior level [tier 3 according to the classification by McKay et al., 2022] and senior level (tier 4) in Norway. Participants were recruited through the teams’ assistant coaches. In total, 10 female players were interviewed: five junior elite players (mean age 16.8 years, SD = 0.96) and five senior elite players (mean age 23.4 years, SD = 2.01), in addition to four of their male coaches (mean age 32.5 years, SD = 3.84) (see Tables 1–3). The players were between the age range of 16–27, trained with the senior team, and the youngest players who combined playing football professionally with attending upper secondary school. All coaches had a minimum of 1 year of experience as a professional coach in the club.

**Table 1:** Participants (junior-elite players).

Pseudonym	Age	Living arrangement	Development status	Senior team experience
Emma	16	Home	Trained regularly with senior team	Few substitutes
Mary	18	Home	Trained regularly with senior team	Few substitutes
Jo	17	Home	Trained regularly with senior team	Trainings
Jennifer	16	Away	Started regularly in recruit team	None
Sara	18	Away	Started regularly in recruit team	None

**Table 2:** Participants (senior-elite players).

Pseudonym	Age in transition	Years since transition	Status
Tara	16–17	5 years	From level 2 club to level 1 club, 3 years ago
Mia	16–17	4–5 years	From junior to senior in level 1 club
Sofia	16–17	5–6 years	From level 2 club to level 1 club, 3 years ago
Louisa	16–17	9–10 years	Transition to level 1 club
Polly	14–15	6–7 years	Transition to level 1 club

**Table 3:** Participants (coaches).

Pseudonym	Age	Education	Responsibilities
Peter	25–30	UEFA B-license, academic education	Role coach, coordination of the players everyday life both related to junior and especially senior players.
John	31–36	UEFA A-license, academic education sport science	Assistant coach, individual follow-up both junior and senior
Mark	31–36	Academic education sport science	Develop players for the senior team, individual follow-up of junior players
Paul	31–36	UEFA A-license, academic education sport science	Educate coaches, develop sport goals and strategies both for junior and senior teams, coordinate collaboration between club and upper secondary school.

### 2.3 Interviews

The data were collected through individual semi-structured interviews. The players were recruited through the coaches in the professional football club. The last author established contact with the club, so that the second and third authors could contact the players and coaches with information about the project (information sheet and consent form) and to schedule the time and place for the interviews. Since the study is part of a larger project on female athletes and their road to elite sports, we combined the two data collections, both performed through interviews. Prior to the interviews, the authors made an interview guide, which included the following topics: experiences with career transitions, stressors, and coach–athlete relations. The interviews were conducted by the second (junior elite players and their coaches) and third authors (junior elite players and former junior elite players) and took place in the teams’ sports arena. The interviews with the junior elite players were performed collaboratively by both the second and third authors. On average, they lasted 38.15 min (SD = 8.78) for the junior elite players, 40.29 min (SD = 10.96) for the senior elite players, and 93.10 min (SD = 35.2) for the coaches. The interviews were audio-recorded and later transcribed verbatim. To ensure confidentiality, all participants were anonymized in the transcriptions.

The interview guide was structured around three primary topics: *Transition experiences* (e.g., “What do you feel are the biggest challenges with the transition to senior, compared to the junior level?”); *Stressors* (e.g., “In which situations do you feel stressed?”); and *Coach–athlete relationship* (e.g., “Have you experienced stress/uneasiness as a result of your relationship with your coach?”). The questions were phrased so that the current players were asked about their present situation, the former players about the situation when they were in the junior-to-senior transition, and the coaches about their overall experiences with players based on their coaching careers.

### 2.4 Data analysis

The data were analyzed using Braun and Clarke’s (2022) six-step thematic content analysis method. We adopted a combined inductive–deductive thematic analysis, with our analysis initially being grounded in the data before drawing on theoretical concepts and extant literature to deepen our analytic interpretations. We specifically used a former study on male players (Rye et al., 2022) as a starting point for our analysis, since both studies were conducted within the Norwegian football context. The data were first analyzed separately by the second author (junior elite players and their coaches) and the third author (junior elite players and former junior elite players). In the first step, the second and third authors transcribed, read, and re-read the interview data to get a general sense of the data material. In the second step, the initial thematic codes were generated through an inductive analysis of the data by the second and third authors, with themes such as “performance stressors” emerging. These two authors discussed the themes and topics together with the first author to reach consensus on the themes based on the two sets of interviews with the players and the coaches. The two authors discussed and agreed on the main themes and topics before proceeding to the next step in the analysis. These analyses were later reviewed by the last author, who acted as a “critical friend,” providing feedback and challenging the reasoning of the second and third authors’ descriptions. In the third step, the first and last authors analyzed the data based on deductive analysis, discussing how the findings should be categorized and structured into higher-order themes, such as “sport stressors.” In the fourth step, systematic work was performed to review the various themes, during which the first and last authors discussed and confirmed if the themes corresponded with the subcategories and codes. In this step of the analysis, the first and last authors discussed the content of the overall themes and agreed on the subthemes and the synthesis of the findings. In the fifth step, the sub-themes and final categories were reviewed and refined, and we ended up with the two main themes: (a) sport stressors and (b) non-sport stressors. In the final sixth stage of the analysis, the report was produced, and several quotes were chosen to reflect the themes in relation to the study’s aim and previous research. During this phase, a draft version of the manuscript was shared with ‘critical friends,’ including members of the Skill and Performance Development in Sports and School (SPDSS) research group at the Norwegian University of Science and Technology.

### 2.5 Study rigor

In this article, rigor is related to the meaningful coherence between the purpose of the study, the procedure, and the findings. Throughout the whole research process, we tried to work actively with Tracy’s (2010) eight criteria for qualitative research quality: worthy topic, rich rigor, sincerity, credibility, resonance, significant contribution, and ethical and meaningful coherence. For example, during the planning of the study, we discussed the focus of the study and the lack of studies on stressors in the junior-to-senior transition among female players (worthy topic), while still building on the theoretical approaches of earlier studies (rich rigor). During the data collection processes, the second and third authors also discussed our results and our interpretations with the last author to ensure peer agreement and validity and further address ethical issues such as anonymity (credibility, sincerity, meaningful coherence, and ethics). We thereby engaged in critical self-reflection to remain aware of potential biases and predispositions that could influence the research process (Tracy, 2010). Due to the potential impact of the study, we also discussed how these findings could impact the coach–athlete relationship during the junior-to-senior transition (resonance) and the same dyad within other sports (significant contribution).

### 2.6 Ethics

Approvals for the study were obtained from the Norwegian Centre for Research Data (reference number 269724 and 217145) before data collection commenced. The first project included junior elite players and their male coaches, while the second project included junior elite players and former junior elite players.

## 3 Results and discussion

The stressors in the junior-to-senior transition were categorized into two main themes: sport stressors and non-sport stressors. Our focus was on the junior players’ experiences of these stressors during the transition, based on interviews with current and former players and their coaches.

### 3.1 Sport stressors

The most prominent stressor identified in the sporting domain was the difference in performance level between the junior and the senior level. This was highlighted by all coaches and players and included higher requirements regarding intensity, mental focus, and technical and tactical skills. This confirms the findings of Gledhill and Harwood (2019), which showed that female athletes during the transition from junior to senior level are met with higher standards regarding performance compared to previous experiences. This was also a prominent theme among all the current junior players, as explained by Jennifer: “In [the old club] I was the best player on the team. But when I got here, almost everyone was better than me.” For the playing up players, these higher standards were considered challenging, both physically and mentally:

“I feel it’s more difficult to do it [adapt] to the recruit team after I’ve been on the senior team. (.) When I’ve kind of been there [the senior team], it’s like I adapt to every day. (.) Also the first training session with the recruit team, maybe I’m a bit tired and a bit busy in my head and stuff like that, so it’s a bit difficult to stay at that [senior] level. For example, I always get it on my left foot when I go forward, and here [recruit team] I get it on my right.” (Mary)

The higher standards regarding performance were also a prominent challenge for the former junior elite players. Louisa explained:

“I also went from dominating every training session in the second division to absolutely not doing it here. So, it was also a challenge in relation to that, and somehow you do not succeed every day when you train with the senior team.” (Louisa)

All players, both former and current junior elite, described similar expectations from themselves, coaches, and teammates, which can be interpreted as a reaction to the competition for playing time and the constant comparison between players (Fortin-Guichard et al., 2022; Platvoet et al., 2020). Some of the players also described the club’s high status as an additional source of stress, since it is one of the best clubs in Norway. These stressors were compounded by entering a new environment with new teammates and coaches, in addition to competing at a higher level than they were used to Swainston et al. (2020). Mary explained it in this manner:

“There is a lot to do with playing in *club* and then with having those expectations on you. So, it’s like that if we lose a game, people think it’s the end of the world because we are *club*.” (Mary)

Due to the differences in level, which was also reinforced by the club’s status, the players were aware of the potential consequences of making mistakes, such as missing opportunities to train with the senior team, which they believed could negatively impact their development (Rye et al., 2022). This was also something two of the former players reflected upon:

“You have to perform all the time. Everyone looks at my performance and likes to compare it against other players performances. (.) You have that pressure on you all the time.” (Tara)

The feeling of being observed and assessed by coaches and other players led to an almost constant pressure to perform. According to Sofia, this was even more prominent during matches:

“I was very nervous before the matches. Because in training it goes quite well, because if I fail, I get another chance. But in matches there’s a lot more at stake.” (Sofia) [sic]

This pressure to perform, especially in a game setting, makes the transition from junior to senior level an emotional process, with a lot of arousal and expectations impacting the players’ well-being. This is in line with findings from Hem et al. (2022), who studied players in professional clubs across Europe, and was also highlighted by some of the coaches in the current study:

“It is natural that they will experience so many emotions. (.) I think it’s expected and natural, and we must do our best to help them in the conversations we have with them and educate them about what it’s going to be like [playing up].” (Peter)

In addition to confirming the athletes’ negative feelings related to performance failure, Peter also mentioned how the age of the athletes made the transition more demanding. Solhaug et al. (2021) found that the youngest players in a men’s football academy in Norway reported significantly higher evaluation stress than older players, and Peter’s statement hints that this also applies to women’s football. Paul described how the players experience stress related to the uncertainty of whether they will be admitted to the senior team over time:

“I experience that they are a bit stressed about how fast it has to go, that they feel like when they play up, if they play up for three weeks, then they feel like they should be part of the senior team. But it may very well be that they actually have to play up for two years, and I feel that they rarely have that much time.” (Paul) [sic]

For some players, this could mean having to withstand hardship over a long period of time:

“You have to deal with running a race where you several times a week, with almost a 100 percent guarantee, will go off the pitch and not have a good feeling that today was successful. And that can be demanding to withstand when you are 16–17 years old.” (Peter) [sic]

In summary, there seem to be several stressors within the sporting domain that are challenging, both physically and mentally. Most of the stressors seem to be related to the increased performance demands at the senior level. These stressors are also reinforced, since the players in the present study belong to one of the best professional clubs in Norway.

### 3.2 Non-sport stressors

While the majority of upper secondary school students in Norway complete their education over 3 years, students in the professional football club follow the same curriculum over 4 years to reduce stressors outside of football. This was labeled crucial for their development by the coaches:

“I think it is very good for the athletes to take upper secondary school over four years instead of three. (.) That gives them a little more time to focus on football with more time and less workload [at school]. (.) The opportunity to have team training instead of school is good because then we can train at ideal times in the morning. (.) It also gives more time for recovery as the days are not so long.” (Mark) [sic]

However, attending these schools often involves moving away from home, which often has been referred to as a major stressor in the research literature (Linnér et al., 2022). This was also something one of the former junior players spoke about:

“The thing about leaving the safety zone. I definitely felt that. Because I had not been away from home before, and it was scary. (…) So, I really felt that the biggest challenge was actually letting go of the safe space [home] and delve into something new.” (Sofia) [sic]

Although the intention and potential benefits of increased focus on their development as football players were clear, the players still pointed out that balancing school and football was challenging, especially during periods with a lot of schoolwork. Furthermore, these challenges arise due to players’ desire to perform well in multiple areas. This can be seen in previous research, which has shown that the prevalence of perfectionism is often high among young athletes. This could also be related to the potential impact of existential anxiety and conflict arising from the tension between ‘wanting to move forward’ and the fear of ‘stepping out of the safety zone’, as found in studies of transitions between clubs in male football (Ransom, 2023). This is clearly reflected in Emma’s quote:

“Those periods with a lot of school. (.) You want to do well on all levels. (.) If you are in training and think too much about the test you have tomorrow, then in a way it can impact your performance, and you do not have as much focus as you should have.” (Emma) [sic]

This was also something Mia, one of the former junior elite players, mentioned while talking about combining football and school: “That [cooperation between school and club] has been important. Because I am someone who wants to do well at school too (.).” Both Emma’s and Mia’s quotes show a wish to perform well in school. More specifically, Emma’s quote also shows how this non-sport stressor could have an impact on their performance, due to a lack of focus. While this could be a problem for both male and female players, it can be argued that female players experience these stressors to a greater extent due to other financial demands and higher risks linked to their future as football players (Andersson and Barker-Ruchti, 2018; Bjerksæter and Lagestad, 2022). This is highlighted in Jo’s quote: “It’s a bit stressful that I need to have as good grades as possible, so that I have a plan B, in case I’m not playing on the senior team by the time I’m finished in school.” This is also in line with previous research indicating that these non-sport stressors occur more frequently among female players (Stambulova and Ryba, 2014), as pointed out by Emma:

“Since we are in women’s football, it’s a bit like that. Or now things have gotten better in Norway, but at least a few years ago it was like that, if you wanted to make a living from football, you kind of had to go abroad. Whereas the boys, in the club they can make a living from it when they are as old as me. That’s not quite the situation I’m in.” (Emma) [sic]

Previous research has found that combining a football career with external pressure from friends and peers makes the transition stressful for both male and female players (Gledhill and Harwood, 2019; Linnér et al., 2022; Rye et al., 2022). In extension of this, McGreary et al. (2021) found in their study of female football players that friends outside of the sport had a positive influence on the players’ development. However, the findings from this study indicated that this can still be problematic, as several players highlighted deprioritizing friends and social events outside of football as challenging. Jo described it as follows:

“All my friends are out partying and stuff like that. While I cannot. (.) I have to go home earlier than everyone else, since there is training the next day. So, there is also a lot of things related to social activities that you cannot attend. You have to prioritize. Prioritize to get as far as possible.” [sic]

Jo’s quote could indicate that her ambition to become a professional football player outweighs the need for a social life. This is similar to findings from male players, where Rye et al. (2022) found that athletes at the professional level were often willing to sacrifice social life for sport. However, having to make this sacrifice could also be challenging regardless of the players’ motivation:

“In a way it could also be a bit challenging that your everyday life when you try to reach the highest level. Every week throughout the whole year, needs to be adapted, to enable you to perform well on every training and every match.” (Mary) [sic]

Finding the optimal balance between sports and everyday life has also been identified as a prominent source of stress for both male and female athletes (McGreary et al., 2021; Swainston et al., 2020). Mark mentioned that dealing with social life in sport is often more challenging for younger athletes, since this is a period when social life, from his perspective, is especially important. This is also reflected in this quote from Mary: “It’s stressful when friends ask if I want to take part in things, but I cannot because I’m supposed to play football.” Research also points out that finding the optimal balance between football and school is a prominent source of stress for both female and male football players (Andersson and Barker-Ruchti, 2018; Linnér et al., 2022). The club in the current study differs from many other clubs in terms of its collaboration with the upper secondary school.

In summary, there seem to be several non-sport stressors that impact both player performance and development. While handling pressure from friends and other peers can be challenging, the most frequent stressor reported was school. The need to prioritize schoolwork may reflect the reality that female players often have fewer opportunities to pursue a professional football career.

## 4 General discussion

Our aim was to contribute insights into junior players’ experiences of sport and non-sport stressors during the junior-to-senior transition in Norwegian football, based on interviews with both current and former junior players and their coaches. Our findings show that current junior players undergoing the transition, former junior players who have successfully completed the transition, and their coaches at both junior and senior levels identify many of the same stressors—similar to findings reported in several studies on male players (Drew et al., 2019). Overall, the findings support the perceived complexity of the transition, where one of the most impactful sport stressors is the fear of making mistakes, despite being in a high-performing environment where skill levels and expectations are higher (Grønset et al., 2024). Among the non-sport stressors, managing a dual career and the school–club cooperation were the most prominent. Despite the described positive relationship between players and coaches in both settings, based on the time players spend with the club and coaches, the coach-player relationship can be crucial in how players handle stressors (McGreary et al., 2021). Although each stressor might be perceived as manageable, they often occur simultaneously, meaning that the players need to handle them at the same time. While we as researchers may categorize stressors as sport and non-sport, the players and the actors in their environment must find ways to handle and address these stressors—often simultaneously. Many of the stressors are also related to being in a high-performance elite context, meaning they persist over time or recur during certain periods of the players’ development (Sæther et al., 2025).

In relation to the introduction of stressors, it is worth exploring in more detail the club’s views on the development of players who spend time with the senior team. It cannot be ruled out that elite football is cynical, considering how few players end up as professionals (Fortin-Guichard et al., 2022) and how only a small number of players successfully handle the transition from junior to senior level (Platvoet et al., 2020). In some environments, the goal of winning matches, which is also present in the studied club given its status in Norwegian football, can come at the expense of the development of individual players. As a counterweight to this cynicism, based on the coaches’ statements, the club emphasizes building good relationships between coaches and players, characterized by openness, trust, and respect. This is in line with the findings of Parpa et al. (2024). This positive experience of the coach–athlete relationship is also reflected in the players’ statements, where it seems that the trust in coaches allows them to communicate about the sources of stress they encounter and their perception of them. A former study of the same junior team environment within the Norwegian professional football club showed that close relationships between the junior elite team and the senior team facilitated skill learning, holistic player development, and mutual learning among coaches within a community of practice (Sæther et al., 2025). This indicates that this close relationship could have a positive impact on players’ experience when training with the senior team while also increasing opportunities to discuss about the stressors the players face during the junior-to-senior transition. Therefore, although it may seem that the players do not necessarily communicate sex-specific stressors, the data suggest that they do express challenges related to the sources of stress that they consider to be most prominent for those playing up. This may serve as effective social support, which can conceivably influence the players’ primary and secondary assessment of sources of stress in a positive way.

The practical implications of the current study may be manifold. Firstly, facilitating arrangements for combining education and football (Gledhill and Harwood, 2015; Ransom, 2023) seems particularly important for female players because of their focus on education as plan B. The studied club’s collaboration with a nearby upper secondary school, as described by both the coaches and the players, appears favorable since it allows players to focus more on sports while reducing psychological and physiological stress through a reduced academic load spread over several years. Given the positive experiences associated with this four-year program, this should be explored in other professional football clubs in Norway. Secondly, it is important to raise awareness among players and coaches about managing expectations and pressure from both non-sport and sport sources of stress. Pressure from coaches, fellow players, teachers, friends, and parents is common, and impact on the players’ sport development and achievements. Sargent Megicks et al. (2023) proposed six principles for holistic development based on the Erasmus+ project ICOACHKIDS: a holistic philosophy of athlete development, stakeholder alignment and support, a climate of care, a long-term learning and development focus, appropriate challenge, and integrated life skill development. This is something that both confederations, regional football federations, and clubs can arrange based on relevant sports psychology knowledge. Thirdly, implementing such educational programs requires that coaches and support staff possess relevant knowledge, as well as the potential inclusion of a sports psychologist. This professional can both assist in athlete conversations about sources of stress and educate coaches and other support staff about important psychological factors that affect athletes’ performance and well-being. This can be carried out under the auspices of the football association, and the inclusion of parents in these educational programs may also be considered.

## 5 Limitations

While this small study revealed several intriguing results, there were some obvious limitations to our design, affecting what these results mean in the broader research context. Most notably, the inclusion of two players with no experience on the senior team when investigating stressors in the junior-to-senior transition was an obvious limitation. Still, the players’ perceptions of the types of stressors they expect to encounter are a vital part of the overall project to which this article belongs. A previous study on age-specific national team players in handball described such expectations as very normative, which could indicate that players have unrealistic expectations about the transition (Bergström et al., 2024). However, these findings showed many similarities between players who had spent time with the senior team and those who had not in terms of both sport and non-sport stressors, indicating that their inclusion still provided valuable insights for the study. Even so, it became clear in the analysis of the data that some of the current junior players both talked more and provided richer descriptions of their experiences compared to others. This highlights the need for future studies investigating sex-specific stressors among female players.

## 6 Conclusion

While the purpose of this study was to gain insights into the sources of stress prominent for female elite football players during the transition from junior to senior level in a professional club in Norway, our findings revealed few sex-specific stressors. Instead, the stressors reported—such as the increased performance demands at the senior level, higher expectations from both players and coaches, and difficulties balancing football with social life outside of sport—mirror those commonly experienced by male players. Even so, the female players described the need for facilitation and support related to education as a plan B to a greater extent than observed in previous studies on male players from professional clubs in Norway. The club’s collaboration with the school on a four-year program also confirms this need, emphasizing the importance of the coach-athlete relationship characterized by mutual respect and trust, helping the players to handle the sport and non-sport stressors. Still, there is a need for future studies on stressors among female athletes in both Norwegian and international football, as well as on how coaches and other actors can facilitate the junior-to-senior transition for female athletes, especially those who emphasize education as a plan B during this process. There is also a need to study players from lower-performing professional clubs to investigate if there are differences in how players perceive stressors compared to those in high-performing clubs.

## Data Availability

The raw data supporting the conclusions of this article will be made available by the authors, without undue reservation.
